# Perfluorinated Compounds in Greenhouse and Open Agricultural Producing Areas of Three Provinces of China: Levels, Sources and Risk Assessment

**DOI:** 10.3390/ijerph13121224

**Published:** 2016-12-10

**Authors:** Yanwei Zhang, Dongfei Tan, Yue Geng, Lu Wang, Yi Peng, Zeying He, Yaping Xu, Xiaowei Liu

**Affiliations:** Key Laboratory of Original Agro-Environmental Quality, Mninistry of Agriculture, Tianjin Key Laboratory of Agro-Environment and Safe-Produt, Agro-Environmental Protection Institute, Tianjin 300191, China; tdf1107921009@163.com (D.T.); gengyue@caas.cn (Y.G.); wanglu@caas.cn (L.W.); ypng_714@126.com (Y.P.); hezeying222308@163.com (Z.H.); nyhjzxtj@163.com (Y.X.)

**Keywords:** PFBA, PFOA, greenhouse agriculture, source analysis, risk assessment

## Abstract

Field investigations on perfluoroalkyl acid (PFAA) levels in various environmental matrixes were reported, but there is still a lack of PFAA level data for agricultural environments, especially agricultural producing areas, so we collected soil, irrigation water and agricultural product samples from agricultural producing areas in the provinces of Liaoning, Shandong and Sichuan in China. The background pollution from instruments was removed and C_4_–C_18_ PFAAs were detected by LC-MS/MS. The concentrations of PFAAs in the top and deep layers of soil were compared, and the levels of PFAAs in different agricultural environments (greenhouses and open agriculture) were analyzed. We found the order of PFAA levels by province was Shandong > Liaoning > Sichuan. A descending trend of PFAA levels from top to deep soil and open to greenhouse agriculture was shown and perfluorobutanoic acid (PFBA) was considered as a marker for source analysis. Bean vegetables contribute highly to the overall PFAA load in vegetables. A significant correlation was shown between irrigation water and agricultural products. The EDI (estimated daily intake) from vegetables should be of concern in China.

## 1. Introduction

Perfluoroalkyl acids (PFAAs) have been widely used in industrial processes and as additives in diverse products, such as clothes and furniture coatings, firefighting foams, paints, metal plating, aviation hydraulic fluids, lubricants and pesticides, due to their unique properties, including surface activity, heat and acid resistance, and water and oil repellency due to their stable carbon-fluorine chains [1,2,3]. While production of perfluorooctane sulfonate (PFOS)-based products was voluntarily halted by North America’s largest producer, The 3M Company, in 2000 [4], large scale production of PFAAs in China began in 2003 [5]. With the shift of manufacturing plants from more industrialized countries to China, the occurrence of perfluorinated compounds (PFCs) in the environment has attracted more attention.

PFAAs are extremely persistent in the environment, since they are not affected by biodegradation or photodegradation [6,7]. Some studies have reported that perfluorooctanoic acid (PFOA) and PFOS are not significantly removed during wastewater treatment and higher levels was actually found after treatment, presumably because of incomplete biodegradation of their precursors [8,9]. Unlike most other persistent organic pollutants, PFOA is water soluble and found in animals in serum rather than fat, and some studies showed that PFAAs could bind to the protein [10]. Because of these properties, PFAAs could be transported over long distances with water, and traces of these substances have been detected even in the Arctic and remote rural areas. Another long range transport pathway for PFAAs transport of their volatile precursors in the atmosphere [3].

Certainly, the agricultural environment has not escaped PFAA pollution. The agricultural environment is polluted by PFAA perhaps due to the long range transport pathway, agricultural water irrigation and use of biosolids-amended soils [11]. Several field investigation papers have reported that PFAAs were widely detected in agricultural products (tea, cereals, salt, sweets, vegetables and fruit items) with PFOA showing a higher concentration [12,13,14,15]. Clark et al. found the concentration of PFOA and PFOS in vegetables from the UK was up to 1000 pg·kg^−1^·ww. The daily intake was far below the existing tolerable levels, but the plant food contribution was equal to that of animal origin food for pefluorononanoic acid (PFNA) and PFOS dietary exposure [16]. Moreover, vegetables and fruit were more important contributors of PFOA than animal foods [15]. There is however a lack of papers reporting the levels of PFAAs in vegetables in China, Zhao et al. found the concentration of PFOA and PFOS were high, and up to 0.84 and 0.42 μg·kg^−1^·ww in Tianjin, respectively, which is much higher than in Europe [17]. The real level of PFAAs in the agricultural environment, especially in agricultural products in agricultural producing areas, is very necessary for the assessment of PFAA level risks to human health.

Many pot experiments with soil and water culture have shown that PFAAs could be translocated from soil or water to plants. Tomato, cabbage, and zucchini could take up PFAAs with the transpiration stream and accumulate them in the leaves [18]. Short-chain PFAAs could be transferred by maize to the shoots with a shoot:root ratio >2 in nutrient solution experiments [19]. Uptake of PFAAs from biosolid-amended soils was also found in carrot, lettuce, radish, celery, tomato, and sugar snap pea, and the bioaccumulation factors (BAFs) were the highest for perfluorobutanoic acid (PFBA) among the perfluorocarboxylic acids (PFCAs) group for all crops [11,20]. PFOA showed higher water solubility than PFOS (PFOA: 3400 > PFOS: 519 mg·L^−1^), and the higher the water solubility, the higher the plant translocation. PFOA showed higher levels in agricultural environments and PFBA showed higher accumulation ability, indicating that the field investigation of PFAAs is very important for PFAA management.

Although PFAAs in remote areas of China have been reported [21], research on the distribution of PFAAs in agricultural producing areas is rather scarce, and only several studies have reported the risk of human exposure for PFOA was lower than the standard value in Europe [12,14], but no systematic report on PFAAs in agriculture producing areas in China has been found. Relatively speaking, the agricultural producing areas in rural areas may serve as the “background area” for PFAA pollution. The sources of PFAAs in agricultural producing areas may simply originate from irrigation water (mostly underground water) and atmospheric precipitation, especially in greenhouse agriculture, where plants are protected and not affected by atmospheric precipitation.

Fluorine chemical industries located in Shandong and Liaoning play important roles in terms of point emission sources and PFAA contamination, dominated by PFOA and PFOS [22]. Two agricultural producing areas in the provinces of Liaoning and Shandong were studied, with Sichuan Province considered for comparison. The aim of the present study was to study the levels of PFAAs in “background areas” of agriculture producing areas; comparison of PFAA levels between greenhouse and open agriculture; and risk evaluation of human exposure for PFOA by dietary vegetable consumption, which might help set standard limits for PFAAs.

## 2. Materials and Methods

### 2.1. Reagents and Standards

Methanol (Fisher, Hampton, NH, USA), ammonium acetate (CH_3_COONHH_4_) (Acros Organics, Hampton, NH, USA) and methyl *tert*-butyl ether (MTBE, J. T. Baker, Coopersburg, PA, USA) were of high performance liquid chromatography (HPLC) reagent grade. Tetrabutylammonium hydrogen sulfate (TBAHS) used as ion-pair reagent was purchased from Sigma (St. Louis, MO, USA). Oasis WAX extraction cartridges (6 cc, 150 mg) were obtained from Waters (Waters Corp., Milford, MA, USA).

Twenty one PFAAs were prepared, including 13 PFCAs and eight PFSAs. The 13 PFCAs included perfluorobutanoic acid (PFBA), perfluoropentanoic acid (PFPeA), perfluorohexanoic acid (PFHxA), perfluoroheptanoic acid (PFHpA), perfluooctanoic acid (PFOA), perfluorononanoic acid (PFNA), perfluorodecanoic acid (PFDA), perfluoroundecanoic acid (PFUnDA), perfluorododecanoic acid (PFDoDA), perfluorotridecanoic acid (PFTrDA), perfluorotetradecanoic acid (PFTeDA), perfluorohexadecanoic acid (PFHxDA), perfluorooctadecanoic acid (PFODA); the eight PFCAs included potassium perfluoro-1-butanesulfonate (PFBS), sodium perfluoropentasulfonate (PFPeS), sodium perfluorohexanesulfonate (PFHxS), sodium perfluoroheptanesulfonate (PFHpS), sodium perfluorooctanesulfonate (PFOS), sodium perfluorononanesulfonate (PFNS), sodium perfluoro-decanesulfonate (PFDS), sodium perfluorododecanesulfonate (PFDoS). Nine ^13^C labeled PFAAs were prepared, including ^13^C_4_-PFBA, ^13^C_2_-PFHxA, ^13^C_4_-PFOA, ^13^C_5_-PFNA, ^13^C_2_-PFDA, ^13^C_2_-PFUnDA, ^13^C_2_-PFDoDA, ^18^O_2_-PFHxS, ^13^C_4_-PFOS. All the native and labeled standards were purchased from Wellington Labs (Guelph, ON, Canada) and all had chemical purities of >98% and isotopic purities ≥99% per ^13^C or >94% per ^18^O. Individual stock standard solutions containing these compounds (1 mg·L^−1^) were prepared in methanol and stored at 4 °C.

### 2.2. Sampling Campaign

**Figure 1:**
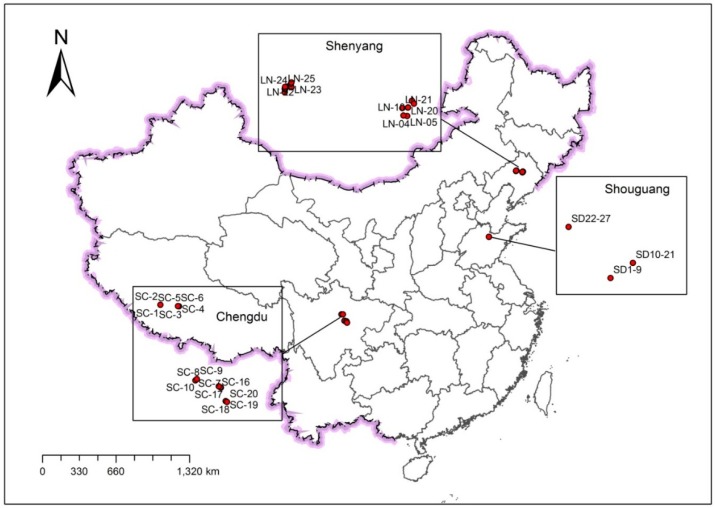
The sampling sites of Shenyang, Shouguang and Chengdu from the provinces of Liaoning (LN), Shandong (SD) and Sichuan (SC), respectively.

The samples were collected from May 2014 to October 2014. Ten, twenty-seven, and twenty-five surface soils (1–20 cm) were collected in the cities of Wenchuan, Dujiangyan and Chengdu from Sichuan Province, Shouguang City from Shandong Province, and the cities of Xinmin and Shenyang from Liaoning Province, respectively (Figure 1). The Sichuan soil samples comprised corn and wheat soils.

At the same place of Liaoning Province where the surface soils were obtained, 25 agricultural products, irrigation water and rhizosphere soil samples were collected. To collect the rhizosphere soil, the plant was first gently shaken to remove the loosely adhering soil as the plant was removed from soil. The remaining adherent soil was separated from the roots as the rhizosphere soil. To collect the irrigation water, local residents were asked about the water provenance and they led us to where we could collect the irrigation water which was shallow groundwater from a well. After pumping for 5 min, the water was collected in 1 L polypropylene (PP) bottles pre-washed with the well water.

All the soils were sampled using polyethylene (PE) hermetic bags that did not contain PFAAs. The soil sample was collected by a 20 cm shovel, and the top soil was sampled from 0–20 cm, and the deep soil was sampled from 20–40 cm. About 0.6 kg of soil was collected by the quartering method from each site, and soil samples of about 3 kg were pooled using five soil samples from about 100 m^2^ of the vegetable-growing areas.

Vegetable samples: Chinese cabbage (*Brasscica rapa*), Romaine lettuce (*Lactuca sativa* L. var. longifolia), baby cabbage (a subspecies of *Brassica pekinensis* (Lour.) Rupr.), celery (*Apium graveolens*), tomato (*Lycopersicon esculentum*), cucumber (*Cucumis sativus*), cabbage (*Brassica oleracea* Linnaeus var. capitata Linnaeus), radish (*Raphanus sativus*), potato (*Solanum tuberosum*), asparagus bean (*Vigna unguiculata*), kidney bean (*Phaseolus vulgaris*). The matching samples including rhizosphere soil and irrigation water were collected around every vegetable site as described above.

All information linking sites, soil number, water number and crop number is listed in Table S1 in the Supplementary Materials. Three travel blanks were checked for sampling events. The travel blank was 100 g wet sediment from the YuQiao reservoir (Tianjin), which had been confirmed to be free of PFAA contamination beforehand analysis. The travel blank were shipped to the field and exposed to the same conditions as the real sample. All dried samples were stored at −20 °C before extraction.

### 2.3. Sample Extraction and Cleanup

#### 2.3.1. Agricultural Products

The extraction of PFAAs from vegetables followed the literature [23] with slight modification. internal standards (^13^C_4_-PFBA, ^13^C_2_-PFHxA, ^13^C_4_-PFOA, ^13^C_5_-PFNA, ^13^C_2_-PFDA, ^13^C_2_-PFUnDA, ^13^C_8_-PFDoDA, ^13^C_4_-PFOHxS, ^13^C_4_-PFOS, 5 ng) were added to the samples and the sample was heated overnight. All the samples were freeze-dried for 24 h. About 0.5 g of dried sample was then homogenized in 5 mL of Mili-Q water, one milliliter of sample was added to a 15 mL PP tube, then 2 mL of 0.5 M sodium carbonate buffer and 1 mL of 0.5 M TBAHS (adjusted to pH 10) were added to the PP tube. After through mixing, the extraction was carried out by the addition of 5 mL of MTBE, and the mixture was shaken vigorously for 40 min. After centrifugation at 8000 r·min^−1^ for 10 min, the supernatant organic liquid was transferred into another PP tube. The extraction procedure was repeated with 3 mL of fresh MTBE, the mixture was shaken vigorously for 20 min and combined with the first fraction. ENVI-Carb^TM^ (150 mg) was added to clean up the samples. After shaking vigorously for 15 min and centrifugation at 8000 r·min^−1^ for 5 min, the supernatant was transferred and evaporated to near-dryness under a gentle stream of high-purity nitrogen gas and then reconstituted with 0.5 mL of methanol. All the final samples in the PP tube were vortexed for 90 s and transferred into an autosampler vial for HPLC-MS/MS analysis.

#### 2.3.2. Water Samples

The Oasis WAX column was activated by methanol with 0.1% ammonium hydroxide, methanol and deinoized water. Internal standards (^13^C_4_-PFBA, ^13^C_2_-PFHx, ^13^C_4_-PFOA, ^13^C_5_-PFNA, ^13^C_2_-PFDA, ^13^C_2_-PFUnDA, ^13^C_8_-PFDoDA, ^13^C_4_-PFOHxS, ^13^C_4_-PFOS, 5 ng) were added to 200 mL of water sample before the samples were accumulated by the WAX column at a speed of one drop per second. After accumulation, 4 mL of sodium carbonate buffer solution (pH 4) was added for purification. The column was eluted using 5 mL of methanol with 0.1% ammonium hydroxide, then the eluted solution was reduced to incipient dryness by a gentle stream of nitrogen gas. After reconstitution in 1 mL of methanol, the solution was analyzed by LC-MS/MS.

#### 2.3.3. Soil Samples

The soil samples were homogenized and sieved (149 μm; USA Standard Testing Sieve, Fisher, Hampton, NH, USA). Internal standards (^13^C_4_-PFBA, ^13^C_2_-PFHxA, ^13^C_4_-PFOA, ^13^C_5_-PFNA, ^13^C_2_-PFDA, ^13^C_2_-PFUnDA, ^13^C_8_-PFDoDA, ^13^C_4_-PFOHxS, ^13^C_4_-PFOS, 5 ng) were added to 2 g of soil sample. After 24 h, 3 mL of methanol was added before ultrasonic extraction for 20 min and concussion for 1 h. After centrifugation at 4200 rpm for 10 min, the supernatant was transferred into a PP tube. The extraction step was repeated and 150 mg of ENVI-Carb^TM^ was added and the mixture was vortexed for 1 min. After centrifugation for 10 min, the supernatant was transferred, reduced to incipient dryness by a gentle stream of nitrogen gas, reconstituted in 1 mL of methanol, and stored at −20 °C for LC-MS/MS analysis.

### 2.4. Instrumentation and Analysis

Quantitative determination of PFAAs were performed on an 30 A liquid chromatography and 8050 triple quadrupole mass spectrometer system equipped with an electrospray interface working in negative ionization mode (Shimadzu, Kyoto, Japan). The separation of PFAAs was conducted on an Acquity UPLC BEH column (2.1 × 50 mm, 1.7 μm, Waters) equipped with a guard column (2.1 × 5 mm, 1.8 μm, Waters). Separation was achieved using 2 mM NH_4_OAc in methanol (B) and 2 mM NH_4_OAc in water (A). A Waters isolater column (2.1 × 50 mm, 5 μm) was installed as a precolumn between the pump and injector. Details about the analytical LC and MS conditions, the parent ions, monitored transitions, and collision energies are provided in the Supplementary Materials (Table S2). All samples were prepared and analyzed in parallel and injected twice per sample.

### 2.5. Quality Assurance and Quality Control

Duplicate samples were analyzed separately. Solvent blank, procedure blank, and sampling blank samples were examined, no PFAAs were detected in these control samples. The LOQ (Limit of Quantification) was identified as ten times signal to noise for each compound, the LOQs of all the PFAA compounds were lower than 0.05 ng·mL^−1^. The recoveries from soil, water, and agricultural product samples ranged from 68.3 to 123% (RSD 0.86%–16.7% of the real samples) except for long carbon chain PFAAs (C_12_ sulfonic acid and carboxylic acids with long chain higher than C_13_) (Table 1).

Quantification was carried out by the internal standard method. The concentration of the standard curve ranged from 0.05 to 10 ng·mL^−1^ with 10 points. The linear coefficients for all PFAA compounds were higher than 0.99 (*r*^2^ > 0.99). When the concentration of ∑PFAAs was higher than the LOD (Limit of Detection) lower than LOQ, the concentration was considered as half of LOQ; when lower than LOD, it was considered zero.

### 2.6. Data Analysis

Statistical comparison (*p* < 0.05, *t*-test) and correlation analysis (Spearman) was conducted using the statistical software package SPSS v17.0 (IBM Corporation, New York, NY, USA), and Origin 8.0 (Originlab Corporation, Redwood City, CA, USA). PCA and OPLS-DA were carried out by MetaboAnalysis (Wishart Research Group, Edmonton, AB, Canada). PCA and OPLS-DA were not software and just analytical method.

## 3. Results and Discussion

### 3.1. Background Pollution of PFAAs from Instruments

Figure S1 shows that background pollution of PFOA. When using polytetrafluoroethylene (PTFE) tubing, significant PFOA pollution was found (Figure S1A). The PTFE tubing was thus changed to stainless steel tubing, and the pollution of PFOA was obviously decreased. To verify the phenomenon, the PTFE tubing was used again and the original pollution showed up again (Figure S1C).

Besides C_8_, background contamination with long chain PFAAs were also found (Figure S2A). To remove this pollution, a mixture of five solvents (methanol, water, acetonitrile, acetone and isopropanol with the same ratio) was used as the needle wash, and the background pollution could be efficiently removed (Figure S2B).

Interestingly, pollution by short chain PFAAs also were found (Figure S3). As reported in the literature, an isolater column from Waters was used that could efficiently remove the background pollution of short chain PFAAs (C_3_ and C_4_) (Figure S3B). At the same time, two C_18_ columns (GL), Shimadzu, Kyoto, Japan) in series were used and when the front one of the two columns was added before the pump after the needle, we found that it could remove the background pollution and showed the same performance as the isolater column from Waters.

### 3.2. The Level of PFAAs in Soil Samples

**Figure 2:**
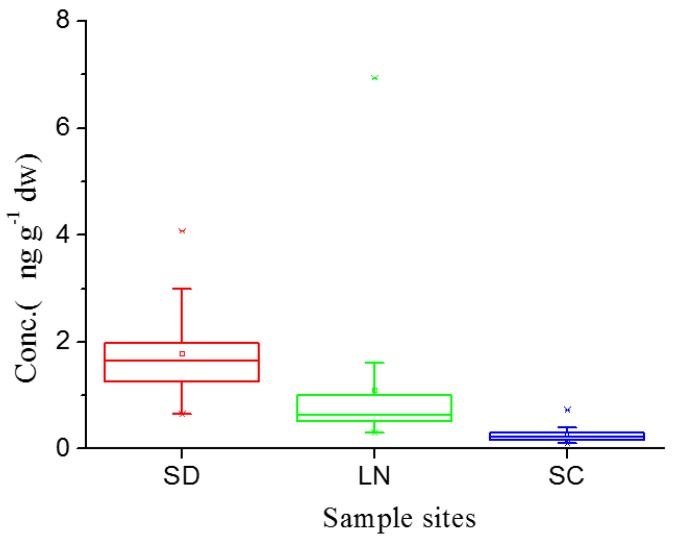
The box plot of concentration of ∑PFAAs in soil samples from Shandong (SD), Liaoning (LN), and Sichuan (SC) province. “□” represents 25%–75%, “*” represents the outliers, “Ⅰ”represents Min–Max.

**Figure 3:**
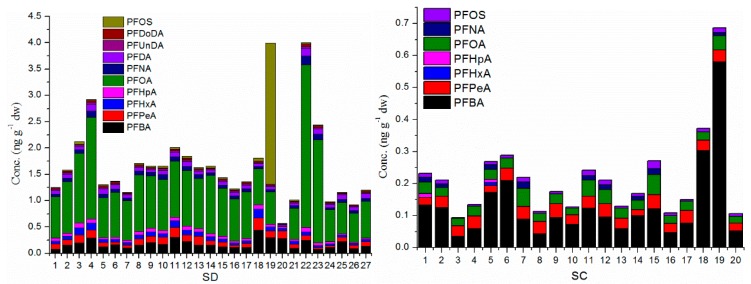

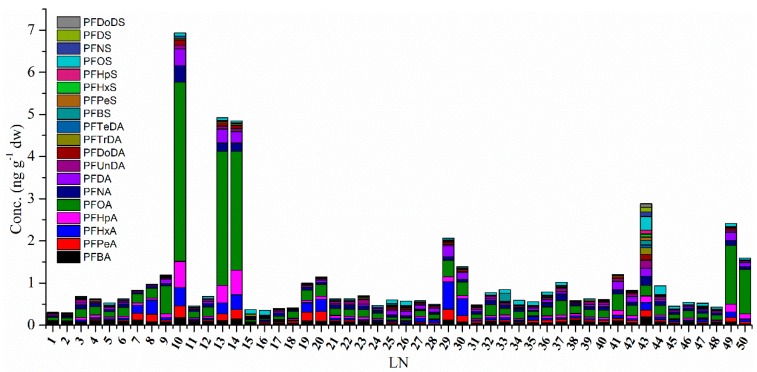
The level of PFAAs in soil samples from Shandong (SD), Sichuan (SC) and Liaoning (LN) provinces. The odd numbers represent the top soil and the even ones represent the deep soil for SC and LN.

Figure 2 and Figure 3 show the concentration of PFAAs in agricultural soil samples from the provinces of Shandong, Liaoning and Sichuan. The concentration of ∑PFAAs was from 0.566 to 4.003 ng·g^−1^·dw with an average concentration of 1.704 ng·g^−1^·dw in Shandong Province. The concentration range of ∑PFAAs was from 0.301 to 4.885 with an average concentration of 1.087 ng·g^−1^·dw in Liaoning Province. The concentration range of ∑PFAAs ranged from 0.094 to 0.686 ng·g^−1^·dw with an average concentration of 0.215 ng·g^−1^·dw in Sichuan Province. As seen from the average concentrations, Shandong showed the highest concentration, followed by Liaoning and Sichuan. The box plot statistically showed the same concentration trend of Shandong > Liaoning > Sichuan. The eastern coastal region showed the higher concentration. Results of previous investigations demonstrated that emissions of PFOS were greater in the more urbanized eastern coastal regions of China [24,25]. Meng et al. reported that the concentration of PFSAs was consistent with urbanization [22].

As seen from a study on 79 surface soil samples from 17 coastal sites in North China, an average concentration of 0.98 ng·g^−1^·dw was reported [22], which was comparable to our value for Liaoning Province, higher than Sichuan Province, and lower than Shandong Province. The concentration of ∑PFAAs in soils around the Koshi River in Nepal ranged from ND (below the detection limit) to 1.78 ng·g^−1^·dw, showing a slightly lower concentration compared to those in China [26]. PFOS and PFOA concentration in soils at former aqueous forming foams training sites ranged from 2.18 to 8520 ng·g^−1^·dw and <0.12–287 ng·g^−1^·dw at military airports Stockholm, Sweden [27]. The concentration of ∑PFAAs in soils around fluorine-based industry parks in China ranged from ND to 3.14 ng·g^−1^·dw [28]. These results provided a conclusive insight that the concentration of ∑PFAAs in agricultural soils showed a higher level compared with the soils from non-point sources, and lower than the levels near point sources.

Figure S4 shows the comparison of PFAAs between greenhouse and open agriculture samples. The concentration in greenhouse samples was significantly lower than that in open agriculture ones in SD (*p* < 0.05). The level in open agriculture samples was slightly higher than that in greenhouse agriculture in LN. The concentration of ∑PFAAs in top soil was slightly higher than that in deep soil (Figure S5). There does not appear to be a significant difference, but a descending trend was noted. The greenhouse environment might protect the soil from PFAA pollution.

### 3.3. The Concentration and Contribution of PFAA Compounds in Soil Samples

**Figure 4:**
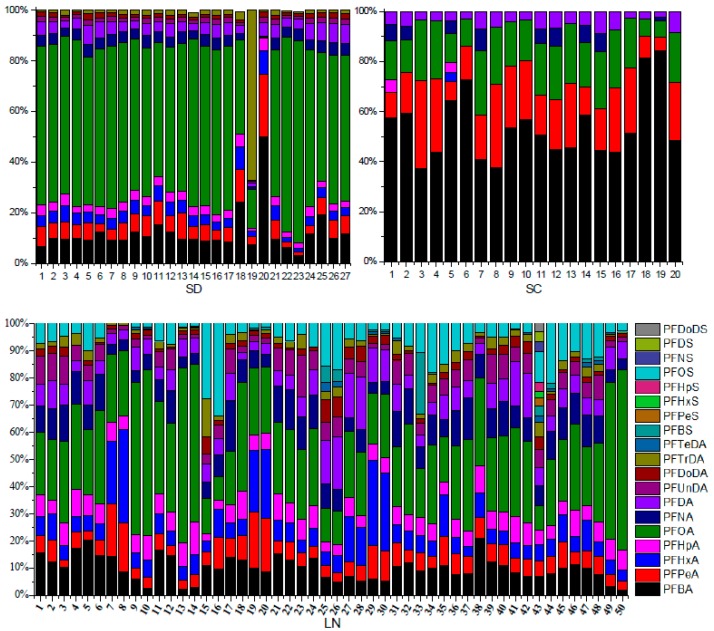
The composition of PFAA compounds in soil samples from Shandong (SD), Sichuan (SC) and Liaoning (LN) provinces. The odd numbers represent the top soil and the even ones represent deep soil for SD and LN.

Figure 3 and Figure 4 show the concentration and ratio of PFAA compounds in all soil samples, respectively. For SD, four sites out of 27 (sites 4, 19, 22 and 23) showed the highest concentrations, and only one site out of 27 (site 20) showed the lower concentration and the remainder of the 27 sites (22 sites) showed concentrations of approximately 1–2 ng·g^−1^·dw. For SC, only two sites out of 20 (sites 18 and 19) showed the higher concentration and the other sites showed concentrations of 0.1–0.3 ng·g^−1^·dw. For LN, eight sites (sites 10, 13, 14, 29, 30, 43, 49 and 50) out of 50 showed the higher concentration, the remaining 42 sites showed concentrations of approximately 0.2–1 ng·g^−1^·dw.

The concentration of PFOS was significantly lower than that of PFOA (*p* < 0.05), and the contribution of PFOS was also obviously lower than that of PFOA. For SD and LN, PFOA showed higher concentrations, however PFBA showed obviously higher concentrations in SC, so maybe the sources of PFAAs were different between coastal and inland areas, and the PFBA and PFBS uses in some industries in SC replaced the uses of PFOS and PFOA as alternatives [29]. Based on the PCA and OPLS-DA analysis (Figure S6), PFBA could be considered as a marker for source analysis, so the higher level concentration of PFBA in SC showed that the sources of PFAAs were different from LN and SD.

PFAA compounds from C_4_ to C_18_ were detected in the present paper, and the number of compounds detected was increasing with the concentration of ∑PFAAs between the three provinces, showing an order LN > SD > SC. Short chain PFAAs (C_4_–C_8_) were detected in all soil samples, the long chain PFAAs (C_9_–C_12_) were detected for PFCAs, but not for PFSAs in SD and SC, and detected both for PFCAs and PFSAs in LN. The super-long PFAAs (PFTrDA and PFTeDA) were detected only in LN, and PFHpDA and PFODA were not detected in any soil samples. The results show that the detection frequency decreased with the increasing carbon chain.

### 3.4. The Concentration of PFAA Compounds in Irrigation Water and Agricultural Products from LN Province 

**Figure 5:**
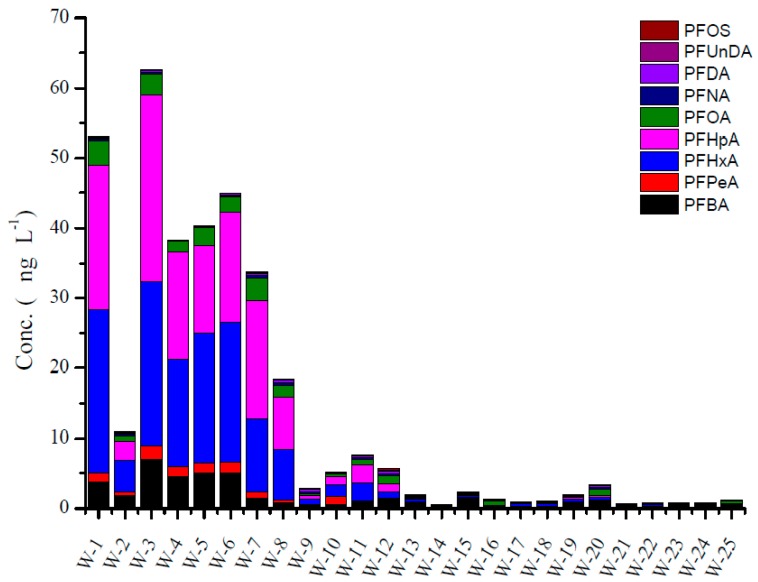
The concentration of PFAA compounds in water samples from LN Province (Liaoning).

The concentration of ∑PFAAs ranged from ND to 8.55 ng·g^−1^ with an average level of 0.98 ng·g^−1^·dw in agricultural products (Figure 5). D’Hollander et al. reported that the range of PFAAs in cereals, salts, sweets and fruit items in four European countries was from ND to 1.09 ng·g^−1^·fw (10.9 ng·g^−1^·dw converted by 90% water content) [12], which was almost consistent with our study. The concentration range of PFAAs in vegetables from the four European countries showed a lower value (ND-1.31 ng·g^−1^·dw). PFCAs were the main group in all agricultural products, whereas PFSAs were not detected in all vegetables and PFOS was not detected with high detection ratio in other references. PFCAs were detected in 76% of all vegetables. PFBA, PFPeA, PFHxA, PFHpA, PFOA, PFNA and PFDA were detected in 40%, 36%, 24%, 36%, 48%, 12%, and 12% in all agricultural products, respectively. PFOA showed the highest detection ratio and PFNA and PFDA showed the lowest detection ratio. The detectable concentrations ranged from 0.03–1.45, 0.06–1.96, 0.04–1.57, 0.02–1.39, 0.005–3.92, 0.03–0.20, 0.03–0.20 ng·g^−1^·dw for PFBA, PFPeA, PFHxA, PFHpA, PFOA, PFNA, and PFDA, respectively. The PFOA showed the highest concentration and the lowest for PFNA and PFDA.

For agricultural products, the different species of vegetables showed obviously different concentrations in 12 species of agricultural products. According to their physical appearance, the vegetables were grouped as shown in Table S3. For stem vegetables (celery) and beans (asparagus bean and kidney bean) the celery and three bean samples showed very high concentrations, while leaf vegetables (Chinese cabbage, lettuce, and baby cabbage), fruiting vegetables (tomato and cucumber), *Brassica* vegetables (cabbage), starchy roots tubers (potato), and root vegetables (radish) showed very low concentrations (Table S3). Only tomato samples from site 7 showed a high concentration.

Figure S7 shows that the bean vegetables contribute highly to the overall PFAA load in vegetables. Maybe the PFAAs can easily to bind with protein [10]. An interesting phenomenon was found in that stem vegetables (celery) contributed most to the PFAA load, showing the highest concentration. The reason for this needs further study. Twenty different types of vegetables were sampled from four countries in the European Union PERFOOD project for PFAA analysis. Herzke et al. found that the leaf vegetables contributed most to the overall PFOA load [14]. Maybe because leaf accumulation was caused by transpiration, perhaps the PFAAs come from the air. One paper on analysis of PFAAs in 43 tea products from South China found that PFOA content was higher than that of PFOS, and the highest concentration of PFOA was 0.25 ng·g^−1^ dry weight [13], compared with the concentrations in stem and bean vegetables.

**Figure 6:**
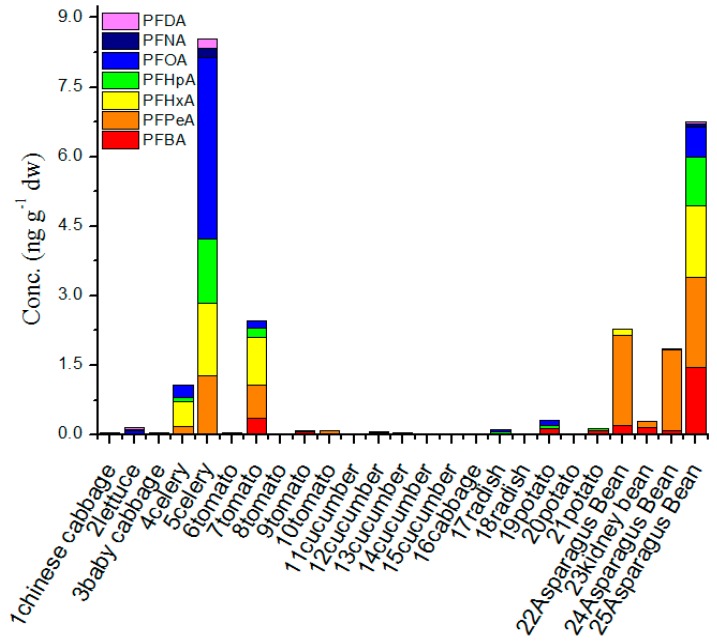
The concentration of PFAA compounds in agricultural products from LN Province (Liaoning).

Figure 6 shows the concentration of PFAA compounds in irrigation water, respectively. The concentration of ∑PFAAs ranged from 0.64 to 62.55 ng·L^−1^ with average value of 13.68 ng·L^−1^ in irrigation water. The detection ratio was up to 100%. PFBA, PFHxA and PFHpA showed the highest contribution, PFOS showed the lowest contribution. Investigations on irrigation water are rather scarce, because the irrigation water is partly from groundwater and river water, so here we compared our results with groundwater and river water. Zhao et al. found PFAAs in rain and snow [30]. Yao et al. reported that the concentration of ∑PFAAs in groundwater from Tianjin and Weifang, China, the concentration of ∑PFAAs was up to ~100 ng·L^−1^ [31]. However, the concentration of ∑PFAAs in groundwater near point sources showed the highest concentration, up to 1,000,000 ng·L^−1^ [32].

Figure S8 shows the concentration correlation analysis between soil, irrigation water and agricultural products for PFOA and PFAAs by the Spearman coefficient determined with the SPSS software. For PFOA, a significant correlation was showed between irrigation water and agricultural products indicating that maybe the PFOA in agricultural products comes from irrigation waters. For PFAAs, no significant correlation was found between soil, irrigation water and agricultural products.

### 3.5. Daily Intake Evaluation of PFOA through Vegetables by the Population in Liaoning Province 

The present study allowed the calculation of dietary intakes for PFOA, due to the relatively high detection frequency in vegetables. For PFOA, a TDI (Tolerable Daily Intake) of 1500 ng·kg^−1^·BW·day^−1^ was given by Europe Food Safety Agency (EFSA), which is that the highest dietary intake value is 90,000 ng·day^−1^, assuing a body weight of 60 kg.

The daily consumption values for vegetables (averaging 308 g·fw·day^−1^ and 232 g·fw·day^−1^ for urban and rural residents, respectively) the were provided by National Bureau of Statistics of China (NBSC 2014). The concentration of PFOA was calculated as 0.022 ng·g^−1^·fw. The calculated estimated dietary intake (EDI) was 6.8 and 5.1 ng for urban and rural residents, respectively. The EDI from urban was higher than rural residents. The dietary exposure estimate ranges of PFOA were 50.6–53.3, 3.24–13.9, 19.0–23.6, 10.9–12.7 ng·day^−1^ for Belgium, the Czech Republic, Italy and Norway, respectively [16]. Compared with the data from Europe, the EDI in the present study showed a lower level. There was no concern about the EDI in vegetables based on the TDI given by EFSA, but the standard level showed differences between USA, Japan and Australia (Table 2). Compared with the TDI from Japan, the EDI in the present paper was the 1:50 times. The TDI contains many sources of PFAAs like dust, water, different food and so on, so the EDI in the present study should be of concern.

## 4. Conclusions

Based on the close relationship between agricultural products and human health, it is very necessary to know the levels of pollutants in the agricultural environment. Through our studies we have found that there appears to be risk of PFAAs from vegetables based on the TDI from Japan. The TDI was different in different countries, and no standard level was found in China, so it is very difficult to assess the real risk of PFAAs from consuming vegetables. It is very necessary to provide a lot of data on EDI from vegetables for the TDI in China. Also it is very important to obtain EDI data from different kinds of vegetables based on bean vegetables that showed the highest contribution to the PFAA load.

## Figures and Tables

**Table 1:** The recaptures and RSDs (real samples) of PFAAs in soils, agricultural products and irrigation water samples.

PFAA	Soils		Agricultural Products		Irrigation Water	
	Recovery (%)	RSD	Recovery (%)	RSD	Recovery (%)	RSD
PFBA	69.6	0.86–9.12	96.3	2.34–5.77	124.7	5.43–6.11
PFPeA	70.9	0.92–5.32	119.1	9.54–12.1	109.8	8.99–9.11
PFHxA	70.1	1.23–8.36	123.3	7.44–9.21	94.2	11.2–15.9
PFHpA	72.6	4.16–9.09	112.3	10.2–14.1	92.8	4.12–8.99
PFOA	76.9	5.62–7.88	92.6	10.1–15.2	86.6	3.56–11.2
PFNA	71.0	2.32–6.89	76.4	2.45–3.66	98.9	3.56–6.88
PFDA	72.2	1.24–12.1	76.1	5.21–10.3	99.1	11.3–16.7
PFUnDA	74.4	8.33–13.6	77.5	10.2–13.4	95.1	12.5–13.1
PFDoDA	70.1	9.23–11.6	71.3	4.21–8.99	72.3	2.34–12.1
PFTrDA	68.1	3.56–15.7	57.8	2.55–6.55	51.8	5.76–13.5
PFTeDA	66.2	12.1–15.4	44.8	4.35–8.99	43.1	12.6–15.7
PFHxDA	43.5	-	34.4	-	35.4	-
PFODA	41.1	-	59.4	-	31.3	-
PFBS	73.9	1.54–3.66	123.0	9.11–10.2	112.4	9.07–10.9
PFPeS	73.4	6.34–2.43	104.5	2.11–5.44	108.8	9.45–13.7
PFHxS	70.3	2.56–7.66	79.4	6.12–9.11	97.7	4.56–12.9
PFHpS	72.4	3.45–8.45	76.4	3.11–8.99	100.1	4.56–14.8
PFOS	70.5	2.67–10.1	80.4	5.66–9.32	109.8	2.45–5.87
PFNS	68.9	2.32–10.5	80.9	9.34–10.6	119.6	9.12–15.6
PFDS	68.3	4.55–8.6	75.1	2.34–9.55	117.7	10.5–15.8
PFDoDS	64.4	-	51.9	-	59.5	-

- Indicates the data was not available.

**Table 2:** The TDI and DI (dietary intake) of PFOA from different countries.

Country	TDI (ng·kg^−1^·BW·day^−1^)	DI (ng·kg^−1^·day^−1^)	Ref.
USA	20	1200	USA EPA
Japan	5.4	324	Japan EPA
Australia	1500	90,000	Australia EPA
EFSA	1500	90,000	EFSA

## Supplementary Materials

The following are available online at www.mdpi.com/1660-4601/13/12/1224/s1, Figure S1: Background pollution of PFOA from PTFE pipelines, Figure S2: The background pollution of long chain PFAAs. A represents the chromatograms before removing the pollution and B represents the chromatograms after removing the background pollution, Figure S3: The background pollution of short chain PFAAs (C_3_ and C_4_). A represents the chromatograms before removing the pollution and B represents the chromatograms after removing the background pollution, Figure S4: The box plot of concentrations of PFAAs from greenhouse and open agriculture in LN and SD province, Figure S5: The box plot for the concentration of PFCs from top and deep layer from LN (Liaoning) and SC (Sichuan) provinces, Figure S6: Principle component analysis (PCA) and orthogonal partial least squares-discriminate analysis (OPLS-DA) analysis for PFAA homologues in soil samples from the provinces of LN, SC and SD (PCA analysis could provide the max different compound between the three provinces, so the compound could be considered as the marker of differentiating source of PFAAs), Figure S7: The relative PFAA distribution in vegetables sub-groups. LV leaf vegetables, SV stem vegetables, FV fruiting vegetables, BV *Brassica* vegetables, RV root vegetables, SR starchy root tubers, BV bean vegetables, Figure S8: The correlation analysis of concentration between soil, irrigation water (water) and agricultural products (prod) by SPSS Spearman correlation for PFOA (top) and PFAAs (bottom), Table S1: The information of linking between soil, irrigation water and agricultural products, Table S2: Details for target PFSAs and PFCAs analyzed by LC/MS/MS, Table S3: Ranges and average of sumPFCA concentrations (ng·g^-1^·dw) in vegetable groups of vegetable items collected in LN.
