# Supplementary material for: Perfluorinated Compounds in Greenhouse and Open Agricultural Producing Areas of Three Provinces of China: Levels, Sources and Risk Assessment
Source: Int J Environ Res Public Health. 2016 Dec 10;13(12):1224. doi: 10.3390/ijerph13121224 (PMC5201365; doi:10.3390/ijerph13121224)
Supplement: Supplementary file 1 [file ijerph-13-01224-s001.pdf]

Supplementary Materials: Perfluorinated Compounds in Greenhouse and Open Agricultural Producing Areas of Three Provinces of China: Levels, Sources and Risk Assessment

Yanwei Zhang, Dongfei Tan, Yue Geng, Lu Wang, Yi Peng, Zeying He, Yaping Xu and Xiaowei Liu

Table S1. The information of linking between soil, irrigation water and agricultural products.

Site	Soil	Water	Products
LN Province			
Site1	Top (LN1) and Deep (LN2)	W-1	1 chinese cabbage
Site2	Top (LN3) and Deep (LN4)	W-2	2 lettuce
Site3	Top (LN5) and Deep (LN6)	W-3	3 baby cabbage
Site4	Top (LN7) and Deep (LN8)	W-4	4 celery
Site5	Top (LN9) and Deep (LN10)	W-5	5 celery
Site6	Top (LN11) and Deep (LN12)	W-6	6 tomato
Site7	Top (LN13) and Deep (LN14)	W-7	7 tomato
Site8	Top (LN15) and Deep (LN16)	W-8	8 tomato
Site9	Top (LN17) and Deep (LN18)	W-9	9 tomato
Site10	Top (LN19) and Deep (LN20)	W-10	10 tomato
Site11	Top (LN21) and Deep (LN22)	W-11	11 cucumber
Site12	Top (LN23) and Deep (LN24)	W-12	12 cucumber
Site13	Top (LN25) and Deep (LN26)	W-13	13 cucumber
Site14	Top (LN27) and Deep (LN28)	W-14	14 cucumber
Site15	Top (LN29) and Deep (LN30)	W-15	15 cucumber
Site16	Top (LN31) and Deep (LN32)	W-16	16 cabbage
Site17	Top (LN33) and Deep (LN34)	W-17	17 raddish
Site18	Top (LN35) and Deep (LN36)	W-18	18 raddish
Site19	Top (LN37) and Deep (LN38)	W-19	19 potato
Site20	Top (LN39) and Deep (LN40)	W-20	20 potato
Site21	Top (LN41) and Deep (LN42)	W-21	21 potato
Site22	Top (LN43) and Deep (LN44)	W-22	22 asparagus bean
Site23	Top (LN45) and Deep (LN46)	W-23	23 kidney bean
Site24	Top (LN47) and Deep (LN48)	W-24	24 asparagus bean
Site25	Top (LN49) and Deep (LN50)	W-25	25 asparagus bean
SC Province			
Site1	Top (LN1) and Deep (LN2)		
Site2	Top (LN3) and Deep (LN4)		
Site3	Top (LN5) and Deep (LN6)		
Site4	Top (LN7) and Deep (LN8)		
Site5	Top (LN9) and Deep (LN10)		
Site6	Top (LN11) and Deep (LN12)		
Site7	Top (LN13) and Deep (LN14)		
Site8	Top (LN15) and Deep (LN16)		
Site9	Top (LN17) and Deep (LN18)		
Site10	Top (LN19) and Deep (LN20)		

Table S1. *Cont.*

Site	Soil	Water	Products
SD Province			
Site1	Top (SD1)		
Site2	Top (SD2)		
Site3	Top (SD3)		
Site4	Top (SD4)		
Site5	Top (SD5)		
Site6	Top (SD6)		
Site7	Top (SD7)		
Site8	Top (SD8)		
Site9	Top (SD9)		
Site10	Top (SD10)		
Site11	Top (SD11)		
Site12	Top (SD12)		
Site13	Top (SD13)		
Site14	Top (SD14)		
Site15	Top (SD15)		
Site16	Top (SD16)		
Site17	Top (SD17)		
Site18	Top (SD18)		
Site19	Top (SD19)		
Site20	Top (SD20)		
Site21	Top (SD21)		
Site22	Top (SD22)		
Site23	Top (SD23)		
Site24	Top (SD24)		
Site25	Top (SD25)		
Site26	Top (SD26)		
Site27	Top (SD27)		

LN: Liaoning; SC: Sichuan; SD: Shandong.

Instrumental Analysis

A mobile phase program based on 2 mM NH_4OAc in methanol (B) and 2 mM NH_4OAc in water (A) at a flow rate of 0.4 mL·min^{-1} was applied, starting at 10% B and increasing linearly to 60% B at 1.5 min, increasing to 85% B at 6 min, and increasing to 100% B at 7.1 min, where it was held for 4 min before going back to the initial condition over 0.1 min. The column was equilibrated with 2% mobile phase B for 4 min between runs. MS (Mass Spectrum) condition: nebulizing Gas Flow: 3 L·min^{-1}, Heating Gas Flow: 10 L·min^{-1}, Interface Temperature 300 °C, DL Temperature 250 °C, Heat Block Temperature 400 °C, Drying gas flow: 10 L·min^{-1}.

Table S2. Details for target PFSAs and PFCAs analyzed by LC/MS/MS.

Compound	Acronym	Ion Transition (m/z)	Q1	CE	Q3
Perfluocarboxylic acids	PFCAs				
Perfluorobutanoic acid	PFBA	213.1/169.0	16	11	17
Perfluoropentanoic acid	PFPeA	263.1/219.0	18	8	24
Perfluorohexanoic acid	PFHxA	313.1/269, 119	22	9	29
Perfluoroheptanoic acid	PFHpA	363.1/319, 169	14	10	12
Perfluooctanoic acid	PFOA	413.1/369, 169	16	11	26
Perfluorononanoic acid	PFNA	463.1/419, 219	18	11	30
Perfluorodecanoic acid	PFDA	513.1/469, 219	20	11	23
Perfluoroundecanoic acid	PFUnDA	563.1/519, 269	22	13	38
Perfluorododecanoic acid	PFDoDA	613.2/569.1, 169	24	13	28
Perfluorotridecanoic acid	PFTrDA	663.2/619.1, 169	26	13	32
Perfluorotetradecanoic acid	PFTeDA	713.2/669.1, 169	20	13	34
Perfluorohexadecanoic acid	PFHxDA	813.2/769, 169	20	14	40
Perfluorooctadecanoic acid	PFODA	913.2/868.9, 169	22	16	32
Perfluorosulfonic acids	PFSAs				
Perfluoro-1-butanesulfonate	PFBS	299/80, 99	22	33	30
Perfluoropenta sulfonate	PFPeS	349/80, 99	27	41	30
Perfluorohexanesulfonate	PFHxS	399/80, 99	30	46	30
Perfluoroheptanesulfonate	PFHpS	449/80, 99	18	48	30
Perfluorooctanesulfonate	PFOS	499/80, 99	19	54	30
Perfluorononanesulfonate	PFNS	549/80, 99	20	50	30
Perfluorodenanesulfonate	PFDS	599/80, 99	20	55	30
Perfluorododecanesulfonate	PFDoDS	699/80, 99	20	55	30
Ionic Labeled PFAAs					
$^{13}C_4$-PFBA	MPFBA	217/172, 59	26	10	18
$^{13}C_2$-PFHxA	MPFHxA	315/270, 120	24	9	30
$^{13}C_4$-PFOA	MPFOA	417/372.1, 168.9	30	11	26
$^{13}C_5$-PFNA	MPFNA	468/423.1, 222.9	18	11	30
$^{13}C_2$-PFDA	MPFDA	515.1/470, 220.1	20	12	23
$^{13}C_2$-PFUnDA	MPFUnDA	565/520.1, 169.1	20	13	38
$^{13}C_2$-PFDoDA	MPFDoDA	615.1/570, 319	24	13	28
$^{18}O_2$-PFHxS	MPFHxS	403/84, 103	15	46	30
$^{13}C_4$-PFOS	MFPOS	503/80, 99	36	50	30

Table S3. Ranges and average of sumPFCA concentrations (ng·g^{-1}·dw^{-1}) in vegetable groups of vegetable items collected in LN.

Groups	Leaf Vegetables	Stem Vegetables	Fruiting Vegetables	Brassica Vegetables	Root Vegetables	Starchy Roots Tubers	Bean Vegetables
	1 Chinese cabbage	4 Celery	6 Tomato	16 Cabbage	17 Radish	19 Potato	22 Asparagus bean
	2 Lettuce	5 Celery	7 Tomato		18 Radish	20 Potato	23 Kidney bean
	3 Baby cabbage		8 Tomato			21 Potato	24 Asparagus bean
			9 Tomato				25 Asparagus bean
			10 Tomato				
			11 Cucumber				
			12 Cucumber				
			13 Cucumber				
			14 Cucumber				
			15 Cucumber				
Conc.	0.05–0.16 (0.09)	1.09–8.55 (4.82)	<MQL-2.46 (0.28)	0.005	<MQL-0.12 (0.06)	<MQL-0.32 (0.15)	0.30–6.76 (2.80)

LV: leaf vegetable, SV stem vegetable, FV fruiting vegetable, BV brassica vegetable, RV root vegetables, SR starchy roots bubers, Be Beans, <MQL less than method quantification limit.

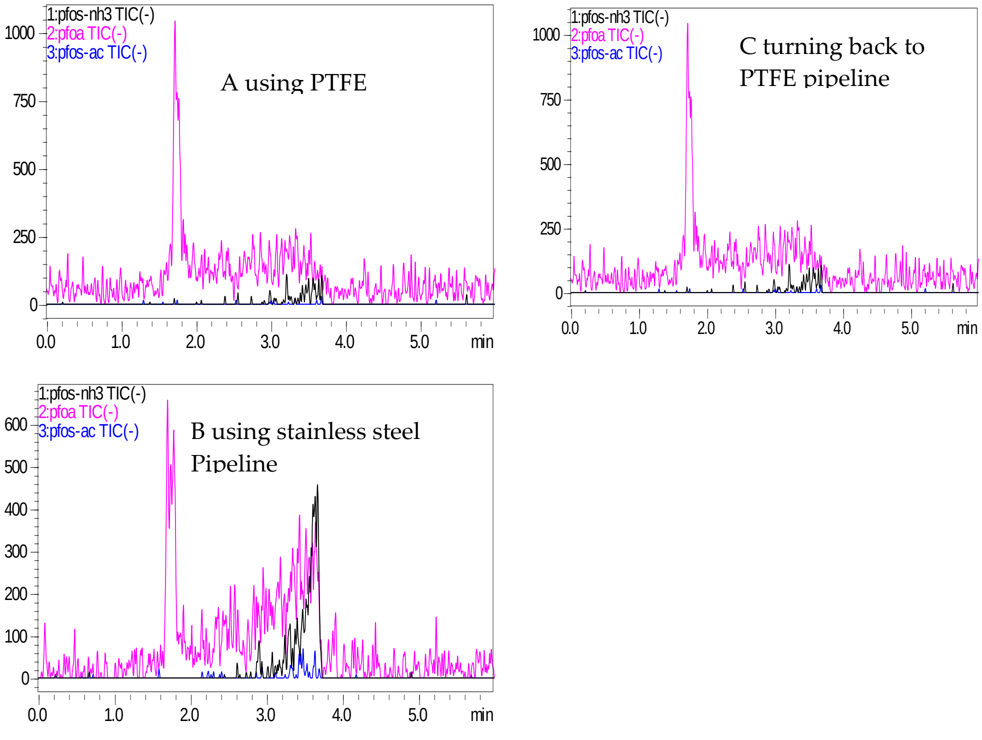

Figure S1. Background pollution of PFOA from PTFE pipelines.

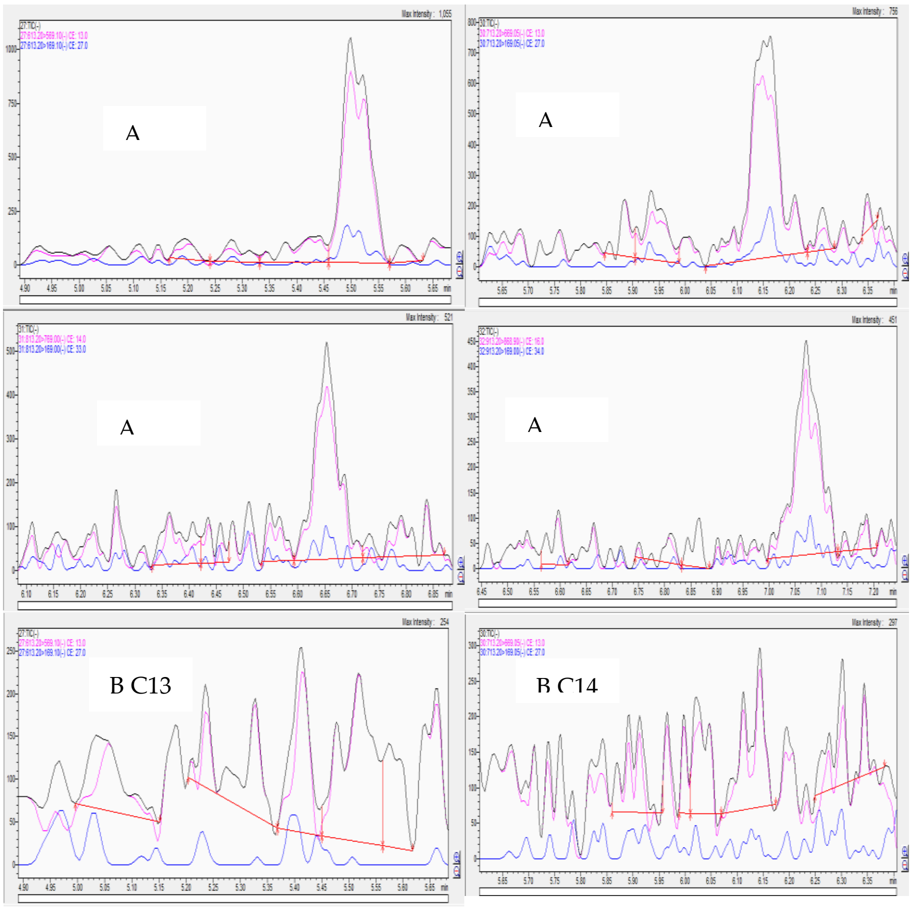

Figure S2. The background pollution of long chain PFAAs. A represents the chromatograms before removing the pollution and B represents the chromatograms after removing the background pollution.

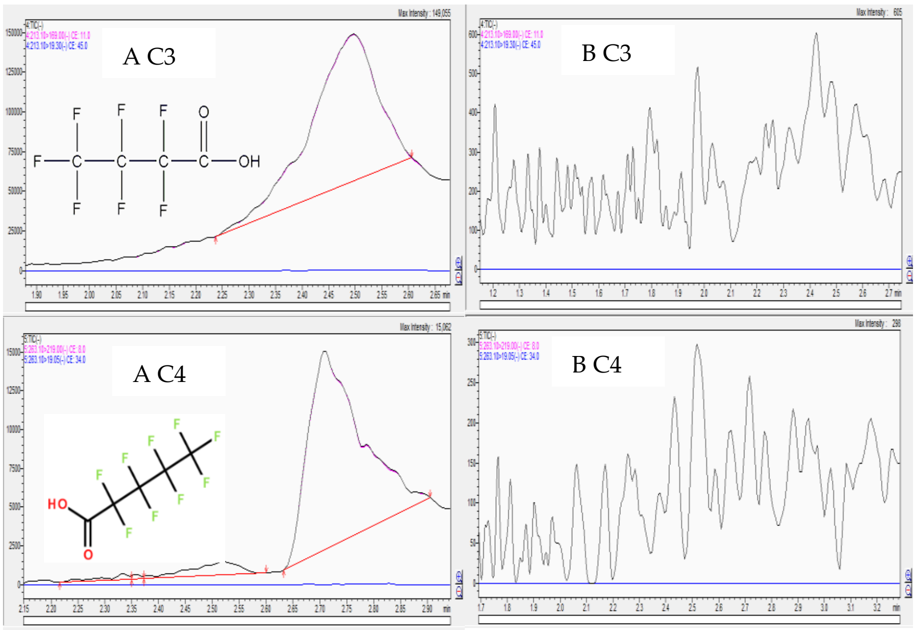

Figure S3. The background pollution of short chain PFAAs (C_3 and C_4). A represents the chromatograms before removing the pollution and B represents the chromatograms after removing the background pollution.

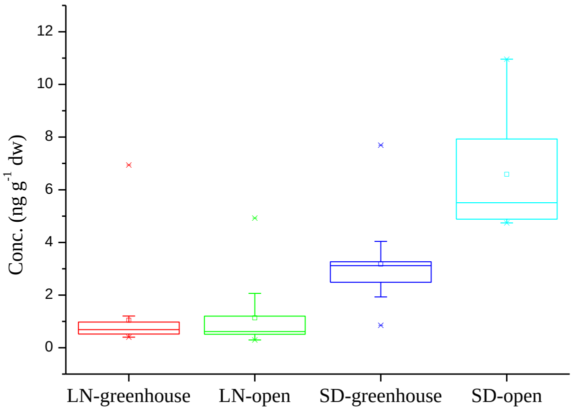

Figure S4. The box plot of concentrations of PFAAs from greenhouse and open agriculture in LN and SD province. "□"represents 25%–75%, "*"represents outlier, " I " represents Min–Max.

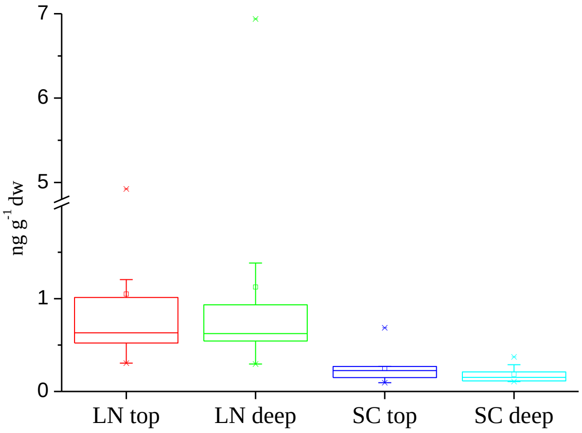

Figure S5. The box plot for the concentration of PFCs from top and deep layer from LN (Liaoning) and SC (Sichuan) provinces. "□"represents 25%–75%, "*"represents outlier, " I " represents Min–Max.

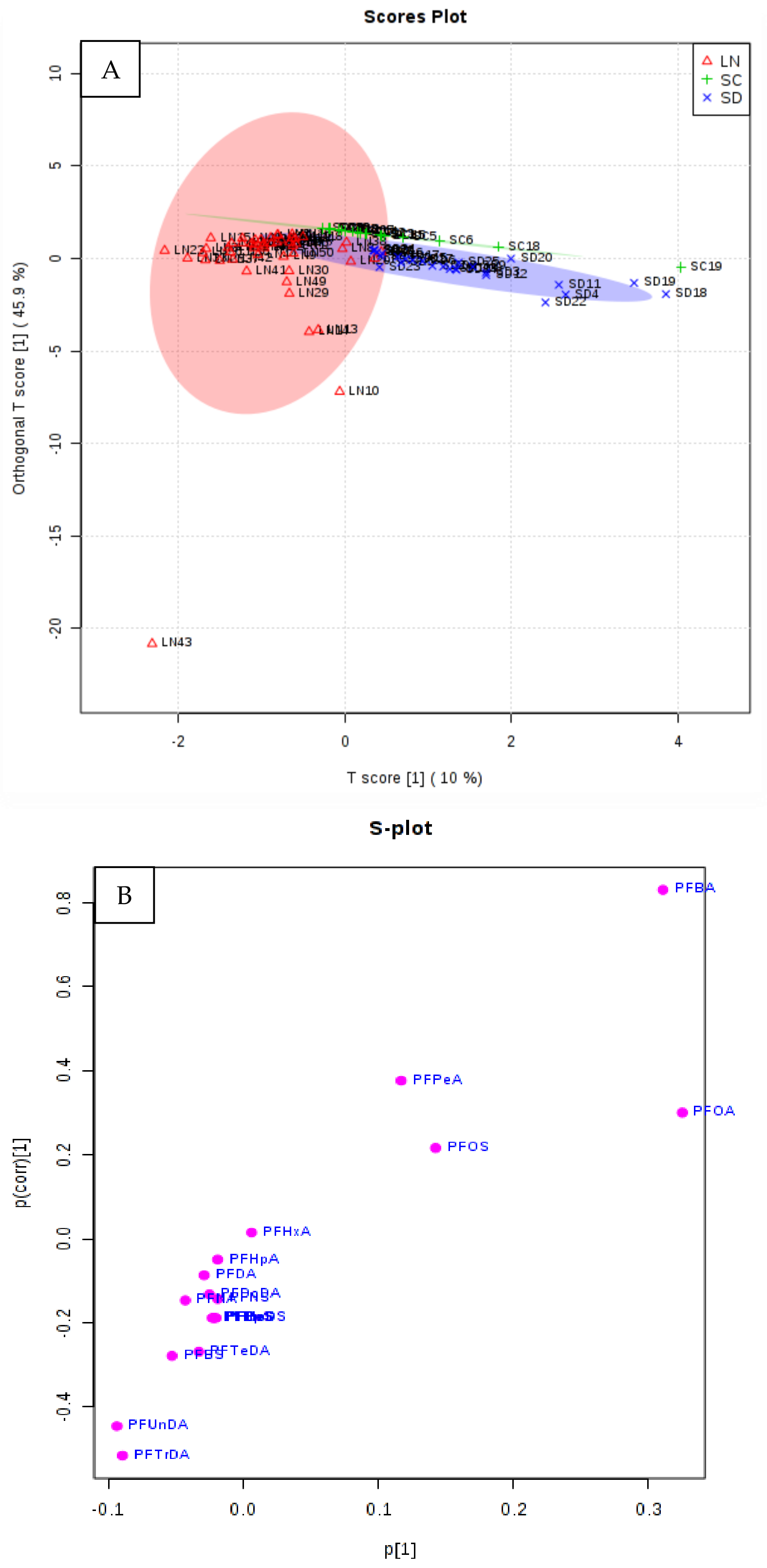

Figure S6. Principle component analysis (PCA) and orthogonal partial least squares-discriminate analysis (OPLS-DA) analysis for PFAA homologues in soil samples from the provinces of LN, SC and SD (PCA analysis could provide the max different compound between the three provinces, so the compound could be considered as the marker of differentiating source of PFAAs). (**A**) represents score plot and (**B**) represents loading plot.

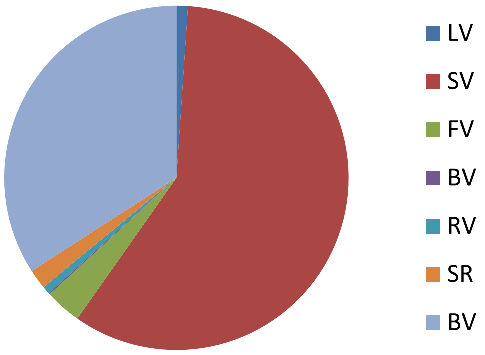

Figure S7. The relative PFAA distribution in vegetables sub-groups. LV leaf vegetables, SV stem vegetables, FV fruiting vegetables, BV *Brassica* vegetables, RV root vegetables, SR starchy root tubers, BV bean vegetables.

Correlations

			prod	water	soil
Spearman's rho	prod	Correlation Coefficient	1.000	.480*	.108
		Sig. (2-tailed)	.	.015	.608
		N	25	25	25
	water	Correlation Coefficient	.480*	1.000	.136
		Sig. (2-tailed)	.015	.	.517
		N	25	25	25
	soil	Correlation Coefficient	.108	.136	1.000
		Sig. (2-tailed)	.608	.517	.
		N	25	25	25

*. Correlation is significant at the 0.05 level (2-tailed).

Correlations

			prod	water	soil
Spearman's rho	prod	Correlation Coefficient	1.000	-.057	.153
		Sig. (2-tailed)	.	.787	.466
		N	25	25	25
	water	Correlation Coefficient	-.057	1.000	.140
		Sig. (2-tailed)	.787	.	.504
		N	25	25	25
	soil	Correlation Coefficient	.153	.140	1.000
		Sig. (2-tailed)	.466	.504	.
		N	25	25	25

Figure S8. The correlation analysis of concentration between soil, irrigation water (water) and agricultural products (prod) by SPSS Spearman correlation for PFOA (top) and PFAAs (bottom).

© 2016 by the authors; licensee MDPI, Basel, Switzerland. This article is an open access article distributed under the terms and conditions of the Creative Commons by Attribution (CC-BY) license (http://creativecommons.org/licenses/by/4.0/).
